# The sodium iodide symporter (*NIS*) as theranostic gene: its emerging role in new imaging modalities and non-viral gene therapy

**DOI:** 10.1186/s13550-022-00888-w

**Published:** 2022-05-03

**Authors:** Carolin Kitzberger, Rebekka Spellerberg, Volker Morath, Nathalie Schwenk, Kathrin A. Schmohl, Christina Schug, Sarah Urnauer, Mariella Tutter, Matthias Eiber, Franz Schilling, Wolfgang A. Weber, Sibylle Ziegler, Peter Bartenstein, Ernst Wagner, Peter J. Nelson, Christine Spitzweg

**Affiliations:** 1 Department of Internal Medicine IV, University Hospital, LMU Munich, Marchioninistrasse 15, 81377 Munich, Germany; 2 Department of Nuclear Medicine, School of Medicine, Klinikum rechts der Isar, Technical University of Munich, Munich, Germany; 3 Department of Nuclear Medicine, University Hospital, LMU Munich, Munich, Germany; 4 Pharmaceutical Biotechnology, Department of Pharmacy, Centre for System-Based Drug Research and Centre for Nanoscience, LMU Munich, Munich, Germany; 5 Division of Endocrinology, Diabetes, Metabolism and Nutrition, Mayo Clinic, Rochester, MN USA

**Keywords:** Sodium iodide symporter, [^18^F]tetrafluoroborate, ^124^I, PET, Glioblastoma, Gene therapy

## Abstract

Cloning of the sodium iodide symporter (NIS) in 1996 has provided an opportunity to use *NIS* as a powerful theranostic transgene. Novel gene therapy strategies rely on image-guided selective *NIS* gene transfer in non-thyroidal tumors followed by application of therapeutic radionuclides. This review highlights the remarkable progress during the last two decades in the development of the *NIS* gene therapy concept using selective non-viral gene delivery vehicles including synthetic polyplexes and genetically engineered mesenchymal stem cells. In addition, *NIS* is a sensitive reporter gene and can be monitored by high resolution PET imaging using the radiotracers sodium [^124^I]iodide ([^124^I]NaI) or [^18^F]tetrafluoroborate ([^18^F]TFB). We performed a small preclinical PET imaging study comparing sodium [^124^I]iodide and in-house synthesized [^18^F]TFB in an orthotopic NIS-expressing glioblastoma model. The results demonstrated an improved image quality using [^18^F]TFB. Building upon these results, we will be able to expand the *NIS* gene therapy approach using non-viral gene delivery vehicles to target orthotopic tumor models with low volume disease, such as glioblastoma.

*Trial registration* not applicable.

**Supplementary Information:**

The online version contains supplementary material available at 10.1186/s13550-022-00888-w.

## Introduction

**Fig. 1:**
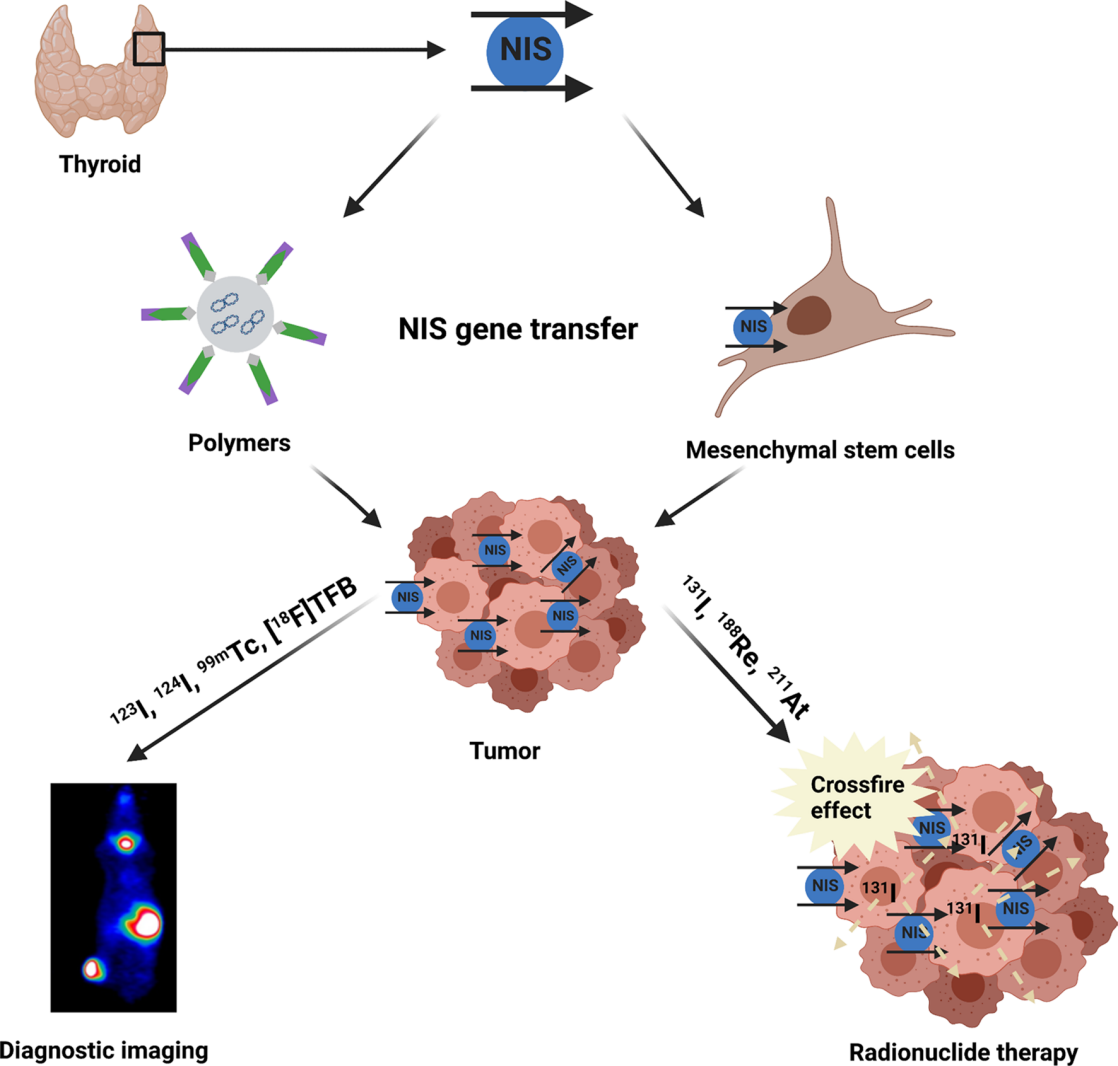
Schematic illustration of NIS and its role in gene therapy. NIS is a powerful theranostic tool for diagnostic imaging and the application of therapeutic radionuclides. The transport of various radiotracers allows non-invasive monitoring of the in vivo biodistribution of functional NIS expression by whole body scintigraphy, SPECT or PET imaging and the application of therapeutically active radionuclides enables cytoreductive effects

The sodium iodide symporter (NIS) is a plasma membrane glycoprotein localized at the basolateral membrane of thyroid follicular cells mediating the active transport of iodide into the thyroid gland as an important prerequisite for the biosynthesis of thyroid hormones (Fig. 1) [1, 2]. NIS-mediated iodide transport can be inhibited by the competitive inhibitors thiocyanate and perchlorate, as well as by the Na^+^Ka^+^-ATPase inhibitor ouabain [3]. Functional NIS expression provides the basis for the diagnostic and therapeutic application of radioiodide that has been widely used in the treatment of differentiated thyroid cancer for 80 years [4]. The cytoreductive effect of targeted NIS-mediated radioisotope therapy is associated with the so called “crossfire effect”, which is the impact of radiation of accumulated radioisotopes in NIS-expressing cells on neighboring non-expressing cells through particle decay [5]. In addition to radioiodide, alternative radionuclides, such as the beta-emitter ^188^Re or the alpha-emitter ^211^At, that are also transported by NIS offer the possibility of higher energy deposition in a shorter time period due to their higher energy and shorter half-life (^188^Re: physical half-life 16.7 h, *E* = 0.764 MeV, path length 23–32 mm; ^211^At: physical half-life 7.2 h, high linear energy transfer 97 keV/µm) as compared to ^131^I (physical half-life 8 d, *E* = 0.134 MeV, therapeutic range 2.6–5 mm), resulting in an enhanced crossfire effect [6–8]. In 1996, N. Carrasco and her team succeeded in cloning of NIS cDNA and thereby provided a new and well-proven dual function tool allowing the establishment of image-guided selective *NIS* gene transfer into non-thyroidal tumors followed by the application of therapeutically effective radionuclides (Fig. 1)—work that was started by the pioneer study by Shimura et al*.* that showed successful restoration of radioiodide accumulation in vitro as well as in vivo after ex vivo stable transfection of transformed rat thyroid cells (FRTL-Tc) with rat NIS cDNA [5, 9, 10].

## Main text

### Non-viral systemic *NIS* gene delivery

An important step in the clinical translation of *NIS* gene therapy of extrathyroidal tumors is the development of effective and safe gene delivery vehicles that allow sufficient and tumor selective NIS expression levels, ideally after systemic vector application. In addition to the option of monitoring and targeting primary tumors, some of these approaches provide options to treat metastases by enhanced targeted delivery of the *NIS* transgene. Non-viral vector systems for targeted *NIS* gene transfer into non-thyroidal tumors are currently under investigation by our group in collaboration with E. Wagner and P. Nelson at the Ludwig-Maximilians-University in Munich and are summarized in this review. Synthetic polyplexes and mesenchymal stem cells can deliver anti-cancer therapies after systemic administration by different targeting strategies. Both systems represent promising platforms with a potential for clinical success.

### Targeted polyplex-mediated and tumor-selective *NIS* gene delivery

Polyplexes are chemically defined carrier systems inspired by viral biology and developed for targeted nucleic acid delivery. These synthetic carriers are designed to overcome some of the current limitations of virus-mediated gene delivery such as immunogenicity, limited nucleic acid binding capacity and difficulty in synthesis and upscaling [11]. Linear polyethylenimine (LPEI) represents the current ‘gold standard’ for synthetic gene delivery systems and is based on polycationic polymers that complex vector DNA through electrostatic interactions. LPEI-targeting and efficiency is refined by the incorporation of polyethylene glycol (PEG) and targeting ligands [12]. PEG shielding lowers the positive surface charge to reduce self-aggregation or aggregation with other biological macromolecules. Furthermore, it protects from immune recognition and provides longer blood circulation [13]. Targeting ligands are for example synthetic peptides that mimic ligands for cell surface receptors overexpressed on cancer cells. Their use can greatly improve the tumor selectivity of gene delivery. The epidermal growth factor receptor (EGFR) is a well-characterized receptor tyrosine kinase upregulated on diverse tumors. The peptide GE11 is a specific allosteric ligand for this receptor [14].

Polyplexes based on LPEI, shielded by PEG and coupled to GE11 (LPEI-PEG-GE11) were employed for systemic *NIS* gene delivery in subcutaneous (s.c.) EGFR overexpressing anaplastic thyroid carcinomas (ATC). ATC is the most aggressive form of thyroid cancer unresponsive to radioiodide therapy. After in vitro characterization of different ATC cell lines, SW1736 with a high EGFR expression level and Hth74 with an intermediate level of EGFR expression were chosen for subsequent in vivo imaging studies. Tumoral ^123^I uptake after systemic LPEI-PEG-GE11 administration was found to be 5.6–7.8% ID/g in SW1736 and 4.5–5.8% ID/g in Hth74 tumors [15]. For ^131^I, this translates to a tumor-absorbed dose of 35.1 mGy/MBq in the SW1736 model and 25.0 mGy/MBq in the Hth74 model [15]. High transduction efficiency and EGFR-specificity of the LPEI-PEG-GE11 polyplex were demonstrated. ^131^I therapy performed in the s.c. SW1736 tumor model showed significant delay in tumor growth and a longer median survival time (42 days) of the therapy group (LPEI-PEG-GE11/NIS + ^131^I) compared to the control groups (28 days for LPEI-PEG-GE11/antisenseNIS + ^131^I and NaCl + NaCl or 18 days for LPEI-PEG-GE11/NIS + NaCl). The data opened the exciting prospect of NIS-mediated radionuclide imaging and therapy of ATC after non-viral reintroduction of the *NIS* gene [15].

The EGFR-targeting approach was then evaluated in an advanced genetically engineered spontaneous mouse model of pancreatic ductal adenocarcinoma (Ptf1a^+/Cre^; Kras^+/LSL−G12D^; Tp53^lox/loxP^ [Kras;p53]). Efficient tumor targeting was demonstrated by [^123^I]NaI-scintigraphy (tumoral iodide uptake: 14.2 ± 1.4% ID/g) and confirmed using three-dimensional high resolution [^124^I]NaI-PET imaging. Following application of [^131^I]NaI, a tumor absorbed dose of 96.5 mGy/MBq was determined. Three cycles of LPEI-PEG-GE11/NIS followed by [^131^I]NaI 48 h later resulted in a significantly reduced tumor growth in this aggressive tumor model [16].

A further evaluation of the EGFR-targeted LPEI-PEG-GE11 polymers was conducted in a mouse model of disseminated colon cancer liver metastases, established by intrasplenic injection of LS174T human colon cancer cells. High levels of NIS-mediated tumoral [^18^F]TFB (tetrafluoroborate) uptake (4.8 ± 0.6% ID) were subsequently measured in mice treated with LPEI-PEG-GE11 (2.2 ± 0.6% ID) as compared to mice injected with untargeted polyplexes (LPEI-PEG-Cys). After administration of [^131^I]NaI, the therapy group showed a significant reduction in hepatic metastases load resulting in extended survival of these mice (15 days post therapy start compared to 8 days for the NaCl + NaCl group or 13 days for the LPEI-PEG-GE11/NIS + NaCl group) [17].

As a next step in the development of polyplex-based *NIS* gene shuttle systems additional sequence-defined polymer backbones containing integrated functional groups were developed, including cationic oligoethano amide cores for enhanced nucleic acid binding and protonable amino acids with buffer function for a higher rate of endosomal escape [18]. In addition, selective targeting using a second important tyrosine kinase receptor was explored. The cMET binding peptide cMBP2 targets the cMET/hepatocyte growth factor receptor (HGFR) that is overexpressed in a majority of cancers [19]. New polymers making use of this biology were evaluated in a s.c. hepatocellular carcinoma (HuH7) xenograft mouse model. High transduction efficiency of cMBP2-PEG-Stp/NIS polyplexes were demonstrated using [^123^I]NaI-scintigraphy: Mice treated with cMBP2-PEG-Stp/NIS polyplexes revealed a significantly higher tumoral iodide accumulation of 6.6 ± 1.6% ID/g as compared to mice injected with untargeted polyplexes (Ala-PEG-Stp/NIS). These results were confirmed in an ex vivo biodistribution study: a perchlorate-sensitive tumoral radioiodide uptake of 3% ID in NIS-tranduced HuH7 xenografts was seen while almost no iodide uptake was measured in tumors of control mice. A tumor-absorbed dose of 41 mGy/MBq for ^131^I was calculated based on the results of the imaging study. A significant delay in tumor growth and prolonged survival was seen in a therapy study after three cycles of polyplex/[^131^I]NaI application [20].

Inter- and intratumoral heterogeneity represent major issues for efficient tumor therapy. To help address this, a dual-targeted polymer was established based on the LPEI-PEG-backbone coupled to both GE11 (EGFR-targeting) and cMBP2 (cMET-targeting). Enhanced tumor targeting of the dual-targeted polyplexes was found as compared to single-targeted polyplexes in an orthotopic HuH7 xenograft mouse model by [^124^I]NaI-PET imaging [21].

### Mesenchymal stem cells as *NIS* gene delivery vehicles

The use of mesenchymal stem cells (MSC) as tumor therapy vehicles is based on their intrinsic tumor-homing capacity [22, 23]. Tumors show an enhanced production of inflammatory cytokines, growth factors and chemokines and thereby drive the active recruitment of MSCs into the tumor microenvironment, where they contribute to the genesis of the tumor stroma [24, 25]. MSCs are well-suited for clinical purposes as they can be easily harvested, amplified and transplanted across the allogenic barrier [22]. Genetically engineered MSCs are promising vehicles for the delivery of therapeutic genes such as *NIS*. The use of engineered versions of MSCs for the treatment of solid tumors are currently being explored in early-phase human clinical trials. One study determining toxicity and efficacy after i.p. administration of engineered MSCs infected with oncolytic measles virus encoding *NIS* as treatment for patients with recurrent ovarian cancer (Clinical trial ID NCT02068794) is being conducted at the Mayo Clinic (Rochester, MN). A second phase I/II trial was conducted at the University Hospital of the LMU built upon our previous studies using autologous MSCs engineered to express the suicide gene thymidine kinase within tumor environments [26].

A series of preclinical studies have demonstrated the potential of CMV (cytomegalovirus) promoter driven MSC-mediated *NIS* gene delivery in xenograft tumor mouse models that have shown successful selective NIS expression in tumors and metastases plus a robust therapeutic response after [^131^I]NaI application [27–29].

These proof-of-concept studies were expanded to an immunocompetent advanced genetically engineered pancreatic ductal adenocarcinoma mouse model described above. Tumoral ^123^I uptake was assessed by [^123^I]NaI-scintigraphy after intravenous MSC application resulting in an impressive level of NIS-mediated iodide accumulation (16.2 ± 2.9% ID) and a tumor absorbed dose of 136.9 mGy/MBq for ^131^I. The tumor selective radionuclide uptake was confirmed by [^124^I]NaI-PET imaging. A significant reduction in tumor growth was seen in the subsequent ^131^I therapy study [30].

A potential side effect of MSC-directed tumor gene therapy is represented by potential MSC homing to normal tissues as part of normal tissue homeostasis that could lead to potential off-target tissue damage. A series of different gene promoters that become activated in response to signaling pathways within tumor microenvironments were evaluated to better control *NIS* transgene expression and enhance the tumor specificity of MSC-based tumor targeting. Hypoxia-inducible factor (HIF) -1 is a key mediator of the cellular response to hypoxia. Hypoxic regions in cancer are more resistant to conventional chemo- or radiotherapy and therefore efficient targeting of those is an important issue in cancer therapy. MSCs engineered with a synthetic HIF-responsive promoter (HIF-NIS-MSC) showed effective transgene induction in vitro under hypoxic conditions using tumor cell spheroid models. In in vivo studies, *NIS* transgene expression was compared between an orthotopic intrahepatic HuH7 mouse model and s.c. HuH7 flank tumors. The maximum ^124^I uptake in the orthotopic tumors was elevated (6.9 ± 0.9% ID/g with a tumor-absorbed dose of 46.8 mGy/MBq ^131^I) as compared to the maximum ^123^I uptake in s.c. tumors (3.9 ± 0.4% ID/g with a tumor-absorbed dose of 26.5 mGy/MBq for ^131^I). These results were confirmed in ex vivo biodistribution studies. The higher tumoral iodide accumulation in the intrahepatic tumors was based on more efficient MSC recruitment due to a more physiologic tumor microenvironment and resulted in a successful ^131^I therapy study. The delay in tumor growth seen in the therapy group (HIF-NIS-MSC + ^131^I) was associated with a reduced tumor perfusion as assessed by contrast-enhanced ultrasound imaging [31].

Activation of the tumor growth factor (TGF-) β/Smad signaling pathway is strongly linked to tumor biology. The use of a synthetic SMAD-based TGF-β-responsive gene promoter to drive *NIS* transgene expression in engineered MSCs (SMAD-NIS-MSC) was evaluated in a series of experimental tumor settings. SMAD-NIS-MSCs induced an ^123^I uptake of 6.8 ± 0.8% ID/g as visualized by [^123^I]NaI-scintigraphy and a tumor-absorbed dose of 28.2 mGy/MBq for ^131^I in a s.c. HuH7 xenograft mouse model. The MSCs were effective in tumor homing and showed a robust TGF-β-induced NIS expression. While the tumor-absorbed dose was lower than that seen in previous studies, the ^131^I therapy study resulted in a stronger therapeutic effect including a significant delay in tumor growth and prolonged survival [32].

New strategies to enhance the tumor-homing properties of MSCs were developed making use of additive effects of combining MSC-mediated *NIS* gene therapy with other treatment options. Potential additive effects could help optimize the therapeutic effectiveness of cancer treatment and overcome tumor resistance. External beam radiation therapy (EBRT) when used in cancer therapy causes extensive tissue damage. EBRT-treated tumor tissues release inflammatory chemokines and growth factors known to be linked to MSC migration [33]. After irradiation of HuH7 cells in vitro, a strong dose-dependent increase in steady state mRNA levels of CXCL8, CXCL12, FGF2, PDGFβ, thrombospondin-1, VEGF and TGF-β1 was found and validated by ELISA. A live cell tracking migration assay monitored by time-lapse microscopy showed that MSCs migrate preferably to supernatant of EBRT-treated HuH7 cells as compared to supernatant from untreated HuH7 cells. MSC migration after EBRT pre-treatment was examined in vivo using *NIS* as a reporter gene. A significant dose-dependent accumulation of radioiodide after i.v. injection of CMV-NIS-MSCs was shown by [^123^I]NaI-scintigraphy. Subcutaneous HuH7 tumors irradiated with 5 Gy revealed the highest ^123^I uptake (9.2 ± 1.5% ID/g) as compared to 2 Gy (7.9 ± 1.4% ID/g) and non-irradiated tumors (5.3 ± 0.8% ID/g). These results demonstrated enhanced tumor homing of MSCs after EBRT treatment of the tumor [34].

The increased TGF-β1 seen after tumor irradiation raised the prospect of applying EBRT prior to injection of SMAD-NIS-MSCS to better control and focus *NIS* transgene expression within the tumor. EBRT enhances the migratory behavior of MSCs, and may also act to amplify SMAD-based promoter activation due to enhanced release of TGF-β1. The combination of focused EBRT (5 Gy) with MSC-mediated systemic *NIS* gene delivery under control of the synthetic TGF-β1-inducible SMAD-responsive promoter was evaluated. [^123^I]NaI-scintigraphy was performed followed by a ^131^I therapy in a s.c. HuH7 xenograft mouse model. Non-irradiated tumors revealed an iodide accumulation of 7.0% ID/g with a tumor-absorbed dose of 52.37 mGy/MBq for ^131^I, while tumors pre-treated with a radiation dose of 5 Gy 24 h before MSC application showed an iodide uptake of 9.8% ID/g and a tumor-absorbed dose of 56.72 mGy/MBq for ^131^I. In the therapy study, the therapy group (5 Gy + SMAD-NIS-MSC ^131^I) showed a pronounced reduction in tumor growth leading to a complete tumor remission in a subset of mice and a dramatically prolonged survival of animals as compared to 5 Gy + CMV-NIS-MSC + ^131^I treated mice or untreated controls. We believe this robust therapeutic effect can be linked to a series of relevant issues: The tissue damage caused by EBRT leads to increased cytokine levels that enhance recruitment of MSCs. The enhanced levels of TGF-β1 further activate *NIS* transgene expression. NIS-based radioiodide treatment causes further tissue damage leading to higher TGF-β1 levels. Thus, a self-energizing cycle may be responsible for the pronounced therapeutic effect seen in this study. The SMAD-responsive promoter may represent a powerful indirect radiation-responsive promoter [35].

Another approach evaluated the combination of regional hyperthermia and MSCs to increase MSC recruitment to the tumor stroma. Hyperthermia is an adjuvant tool in multimodal treatment approaches and is used to enhance therapeutic efficacy. Pleiotropic effects on malignant cells, such as reduction of DNA repair, heat shock protein (HSP) production and modulation of inflammatory cytokines are thought to help trigger an antitumor immune response. This biology also suggested a potential basis for combining MSC based *NIS* gene therapy with local hyperthermia. Hyperthermia of HuH7 cells in vitro resulted in an increased production of immunomodulatory factors and in a live cell tracking migration assay MSCs showed directed chemotaxis towards the supernatant of heat-treated cells as compared to non-treated HuH7 cells. The enhanced migration of CMV-NIS-MSCs in vivo to heat-treated s.c. HuH7 tumors was demonstrated by [^123^I]NaI-scintigraphy (8.9 ± 1.1% ID/g for tumors heated at 41 °C as compared to 5.4 ± 0.5% ID/g for 37 °C). A ^131^I therapy study resulted in significantly enhanced efficacy by combining CMV-NIS-MSC-based *NIS* gene delivery with regional heat treatment 24 h later and a [^131^I]NaI injection 48 h later [36].

In a subsequent series of experiments, MSCs were engineered with a heat-inducible HSP70B promoter allowing tumor-specific, time- and temperature-dependent NIS expression. Optimal promoter activation was evaluated using [^123^I]NaI gamma imaging. Iodide application 12 h after 41 °C heat treatment revealed the highest tumoral uptake (9.7 ± 2.3% ID/g as compared to 6.8 ± 1.9% ID/g in 37 °C controls). This most optimal application regime was then evaluated in a ^131^I therapy study where the therapy group showed a reduction in tumor growth and an extension in survival length [37].

### The role of NIS in advanced imaging modalities

As demonstrated in the previous sections, *NIS* is a powerful theranostic gene that allows the efficient monitoring of molecular therapies after application of radionuclides [9]. *NIS* also has many features of a well-suited reporter gene: It is a naturally occurring protein originating from thyroid follicular cells and is non-immunogenic and non-toxic to cells [5]. As iodide accumulation only occurs in living cells, functional NIS activity is associated with cell viability [5]. The active transport of substrates leads to an accumulation of radiolabeled substrates and concentrates the signal. Thus, the detection sensitivity is higher as compared to a reporter that simply binds its substrate stoichiometrically [38]. NIS translocates various substrates and thereby makes various standard nuclear medicine imaging modalities suitable for localizing NIS-positive cells. The active transport of ^123^I, ^125^I, ^131^I, ^99m^Tc and ^188^Re facilitates gamma scintigraphy and single-photon emission computed tomography (SPECT). Additionally, ^131^I and ^188^Re are therapeutically effective radionuclides through their beta decay. Planar scintigraphy or SPECT have been used as the core technologies of molecular imaging of NIS in the clinic. However, the imaging of functional NIS expression by PET allows the prospect of improved resolution, sensitivity and effective quantitative analysis [39]. As positron emitter, ^124^I is the best known and most often applied tracer for NIS-mediated PET imaging in the preclinical and clinical setting. However, ^124^I has several disadvantages for the routine diagnostic clinical use: It has a relatively long half-life of 4.2 days, a low positron yield (23%), high positron energy and additional high energy gamma emissions (> 500 keV) that result in high radiation exposure and image quality degradation [40]. Further, the complexity of its production leads to high costs and limited availability.^124^I is currently commercially available from only a few sites in Europe. The situation is compounded by the fact that Perkin Elmer stopped its distribution in 2019 and only very few reliable ^124^I sources such as DSD-Pharma remain. Through organification of iodide in the thyroid, the tracer is made less available for non-thyroidal targeted cells due to this “thyroid-sink” effect. To reduce this impact and to avoid toxic thyroidal off-target effects, patients may be pre-treated with L-thyroxine to downregulate the TSH-dependent thyroidal NIS expression and thus lower iodide uptake in the thyroid [41]. A novel tracer for NIS-based PET imaging was recently established. ^18^F-Tetrafluoroborate (TFB) has been introduced as a potential alternative to ^124^I showing several advantages for routine diagnostic use based on the radiochemical and physical properties of ^18^F [39, 43]. [^18^F]TFB possesses advantages over ^124^I due to its shorter half-life (110 min vs. 100 h), branching ratio (97 vs. 23%) and especially the lower positron energy (*E*_max_; 0.634 vs. 2.14 MeV) resulting in a clearer and less ‘blurred’ PET image [39, 40, 42, 43]. TFB is a fluorine-containing ion that is structurally comparable to pertechnetate, a substrate of NIS as shown by electrochemical studies [43]. [^18^F]TFB is trapped, but as is seen with [^99m^Tc]pertechnetate, it is not organified by the thyroid. As a nonorganified NIS tracer, it shows a biodistribution similar to that seen with [^99m^Tc]pertechnetate with a physiological tracer accumulation in the thyroid, stomach, salivary glands, with mainly renal secretion, and is pharmacologically and radiobiologically safe in humans [40, 44, 45]. [^18^F]TFB is a very promising iodide analogue as shown in a series of preclinical animal models and in the clinical setting in healthy individuals as well as thyroid cancer patients [40, 42, 44–48]. In a preclinical study a higher absolute [^18^F]TFB uptake was observed in non-thyroidal NIS-expressing tissues as compared to iodide due to the lack of metabolic entrapment of [^18^F]TFB in the thyroid resulting in a higher availability of [^18^F]TFB to extrathyroidal NIS-expressing organs [49]. In addition, [^18^F]TFB can deliver excellent target-to-background ratios in contrast to the image quality of ^124^I. It shows a high signal-to-noise ratio within ~ 1 h post injection, allows more optimal imaging times for patients and has less radiation exposure for the patient [42]. In a preclinical comparative imaging study using a NIS-expressing orthotopic xenograft breast cancer mouse model, the pharmacokinetic differences between the PET tracer [^18^F]TFB and the SPECT tracer [^123^I]iodide were evaluated, which play a crucial role for imaging performance regarding the imaging sensitivity [49]. The authors found a faster and more complete clearance of [^18^F]TFB from the blood paired with faster tumoral uptake which led to higher target-to-blood ratios as compared to ^123^I and importantly allowed imaging of small NIS-expressing metastases, which were not detectable with routine metabolic [^18^F]FDG-PET. The clinical implementation of [^18^F]TFB has lagged, despite its superiority over ^124^I. To date only a small cohort of individuals have been imaged with [^18^F]TFB [40, 42, 45, 46, 50]. In addition, the short half-life and especially the different pharmacokinetics of [^18^F]TFB compared to iodine do not allow reliable radiation dosimetry extrapolation using [^18^F]TFB for radioiodine treatment. Due to improved image quality, [^18^F]TFB-PET imaging allows expansion of gene therapies that employ *NIS* as reporter gene in low volume tumor models, such as metastatic disease or glioblastomas. Additionally, the potential overlap of the signal seen in these low volume tumors with the organs that physiologically express NIS can be circumvented by improved delineation of signals.

### The role of NIS-imaging in glioblastoma

Glioblastoma (GBM) is the most common primary brain tumor with a poor prognosis and mainly palliative therapy concepts [51]. As a highly complex tumor that exploits several mechanisms to evade therapy, novel treatment strategies for GBM are urgently needed [51]. One reason of restrictions in the effective treatment and detection of GBM is the blood–brain-barrier (BBB) that can block radiotracers and gene vectors. NIS-mediated radionuclide imaging and therapy does not require complex radiolabeling procedures and the small sized radionuclides used are able to penetrate the BBB and diffuse into the tumor [52].

Several preclinical studies have demonstrated the potential application of NIS for glioma imaging and therapy. A study by Cho et al*.* used a rat model with intracerebral F98 gliomas that had been retrovirally transduced with human NIS. The authors showed the possibility of non-invasive glioma imaging by [^99m^Tc]pertechnetate- and [^123^I]NaI-scintigraphy followed by an increased survival time of rats after ^131^I therapy [53]. Guo et al*.* published imaging and therapy experiments with ^188^Re and ^131^I in mice bearing xenografted tumors after injection of the human glioma cell line U87 that was transfected with a recombinant lentiviral vector containing human NIS into the right armpit [52]. In vivo imaging results showed ^188^Re/^131^I accumulation in the NIS-containing tumors as assessed by gamma camera imaging and an effective decrease in tumor volume was achieved in mice receiving ^188^Re or ^131^I as compared to untreated control mice. In another study, using one of the most extensively explored oncolytic viruses for *NIS* gene transfer, Opyrchal et al*.* reported effective [^123^I]NaI or [^99m^Tc]pertechnetate gamma camera or microSPECT/CT imaging of s.c. and orthotopic murine glioblastoma xenografts after intratumoral infection with measles virus encoding NIS (MV-NIS) to induce NIS expression in brain tumor tissue. Combined radiovirotherapy with MV-NIS and ^131^I resulted in an improved antitumor activity and survival as compared to virotherapy alone in both glioma settings [54].

**Fig. 2:**
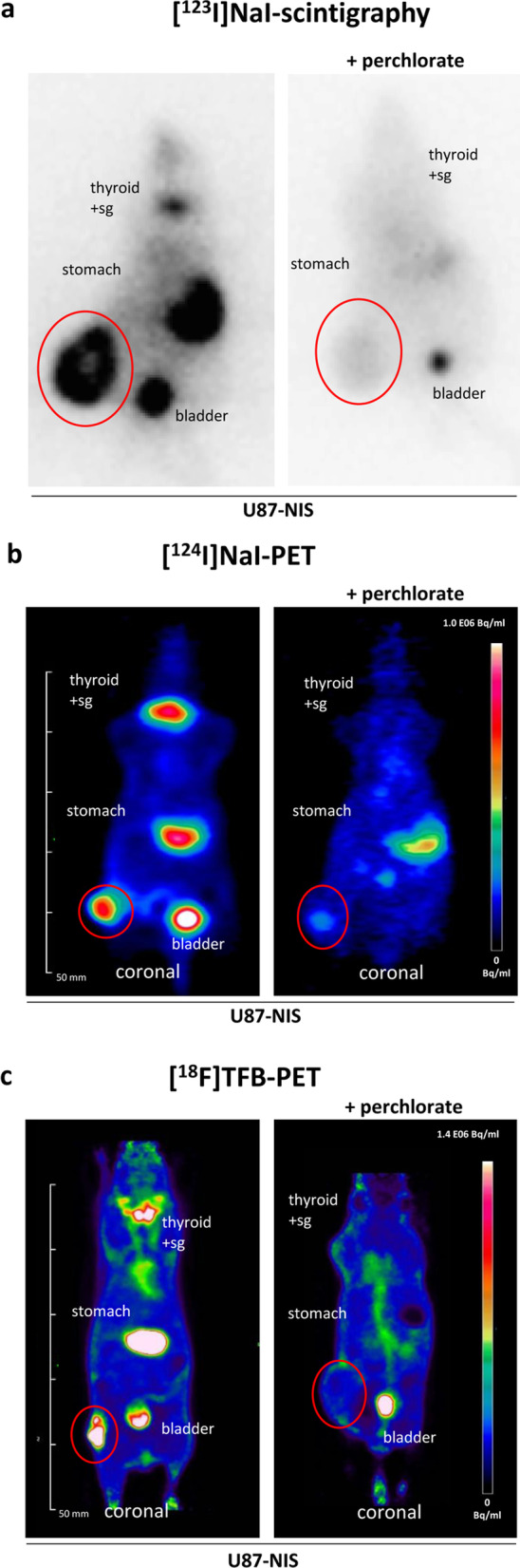
NIS-mediated in vivo imaging of mice bearing subcutaneous U87-NIS tumors. **a** Planar gamma camera imaging showed NIS-specific tumoral ^123^I uptake of 9.4% ID/g tumor (n = 2; + NaClO_4_ n = 1) 1 h after intraperitoneal application of 18.5 MBq [^123^I]NaI. **b** PET scans revealed 4.8 ± 1.1% ID/mL ^124^I accumulation in the tumor (n = 5; + NaClO_4_ n = 3). **c** [^18^F]TFB-PET scans resulted in a maximum tumoral [^18^F]TFB accumulation of 7.1% ID/mL (n = 1, + NaClO_4_ n = 1). Presented PET images show sectional planes (coronal orientation) 1 h after i.v. tracer injection of 10 MBq. Tracer uptake of the tumors was blocked upon treatment with the NIS-specific inhibitor perchlorate. Tumors are circled in red; sg, salivary glands

The advantage of PET, in contrast to scintigraphy or SPECT, is the potential detection of low volume GBM lesions with relatively low NIS expression levels when systemic gene transfer approaches are used [38]. Preclinical imaging studies with [^18^F]TFB as a PET tracer were performed in athymic mice bearing human NIS-expressing C6 glioma s.c. xenografted tumors yielding an avid NIS-mediated tumoral [^18^F]TFB uptake [47]. Recently, a small imaging series by our group was performed using the human U87 glioma cell line stably transfected with a NIS expressing plasmid (CMV-NIS-pcDNA3) (U87-NIS) to track the tumoral NIS expression of s.c. and orthotopic brain tumors by a direct comparison of ^124^I and [^18^F]TFB as radiotracers for small animal PET imaging. The localization of NIS protein on the cell membrane of the U87-NIS cells and its active transport of iodide was confirmed by immunocytochemistry and [^125^I]iodide uptake assays in vitro (Additional file 1: Fig. S1). The U87-NIS cells were used in a s.c. model. Stably NIS expressing U87 tumors revealed a pronounced iodide accumulation (Fig. 2a) and endogenous NIS-mediated ^123^I uptake was observed in stomach, thyroid and salivary glands as well as in the urinary bladder due to renal excretion.

In a second group of mice, NIS-based radionuclide biodistribution was investigated using three-dimensional preclinical PET scanners after intravenous injection of [^124^I]NaI or [^18^F]TFB. The results showed a high accumulation of the NIS PET tracers in U87-NIS tumors (Fig. 2b, c). With a simple and effective in-house synthesis of [^18^F]TFB based on the protocol of Phil Blower’s group from the King’s College in London, we were able to achieve a radiochemical yield of 15% (starting activity of 5 GBq) and a purity of over 97.5% as assessed by radio-thin-layer chromatography [44]. To demonstrate that the radiotracer uptake in U87-NIS tumors was NIS-mediated, mice were additionally treated with the competitive NIS-specific inhibitor perchlorate. In these animals the physiological signal of endogenously NIS-expressing organs (thyroid, mammary glands, salivary glands, stomach) as well as tumoral uptake was effectively blocked.

**Fig. 3:**
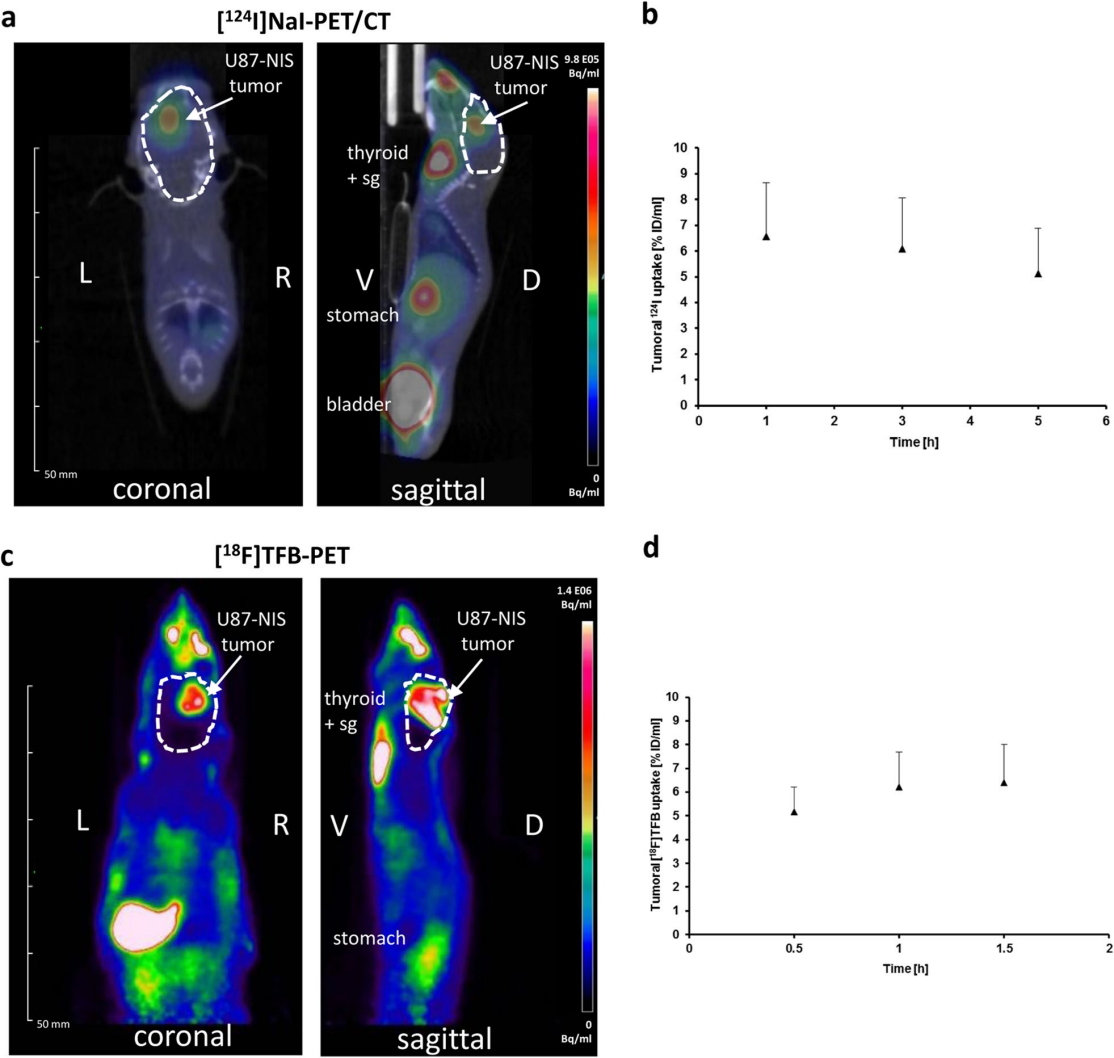
NIS-mediated in vivo small-animal PET imaging of U87-NIS brain tumors. Comparison of U87-NIS brain tumor detection by [^124^I]NaI- and [^18^F]TFB-PET. **a**, **c** Sagittal and coronal planes of [^18^F]TFB-PET and [^124^I]NaI-PET/CT scans are displayed. The brain areas are circled in white and tumoral tracer uptake was seen for both radionuclides (arrows). Low level of bone accumulation indicate a minimal level of residual free fluoride. **b**, **d** Quantification of serial PET imaging representing the efflux of tumoral ^124^I and an increase of [^18^F]TFB in the tumor (n = 5 each). Representative pictures show sectional planes of the 1 h time point after i.v. tracer injection (10 MBq). Results are expressed as mean ± SEM

**Fig. 4:**
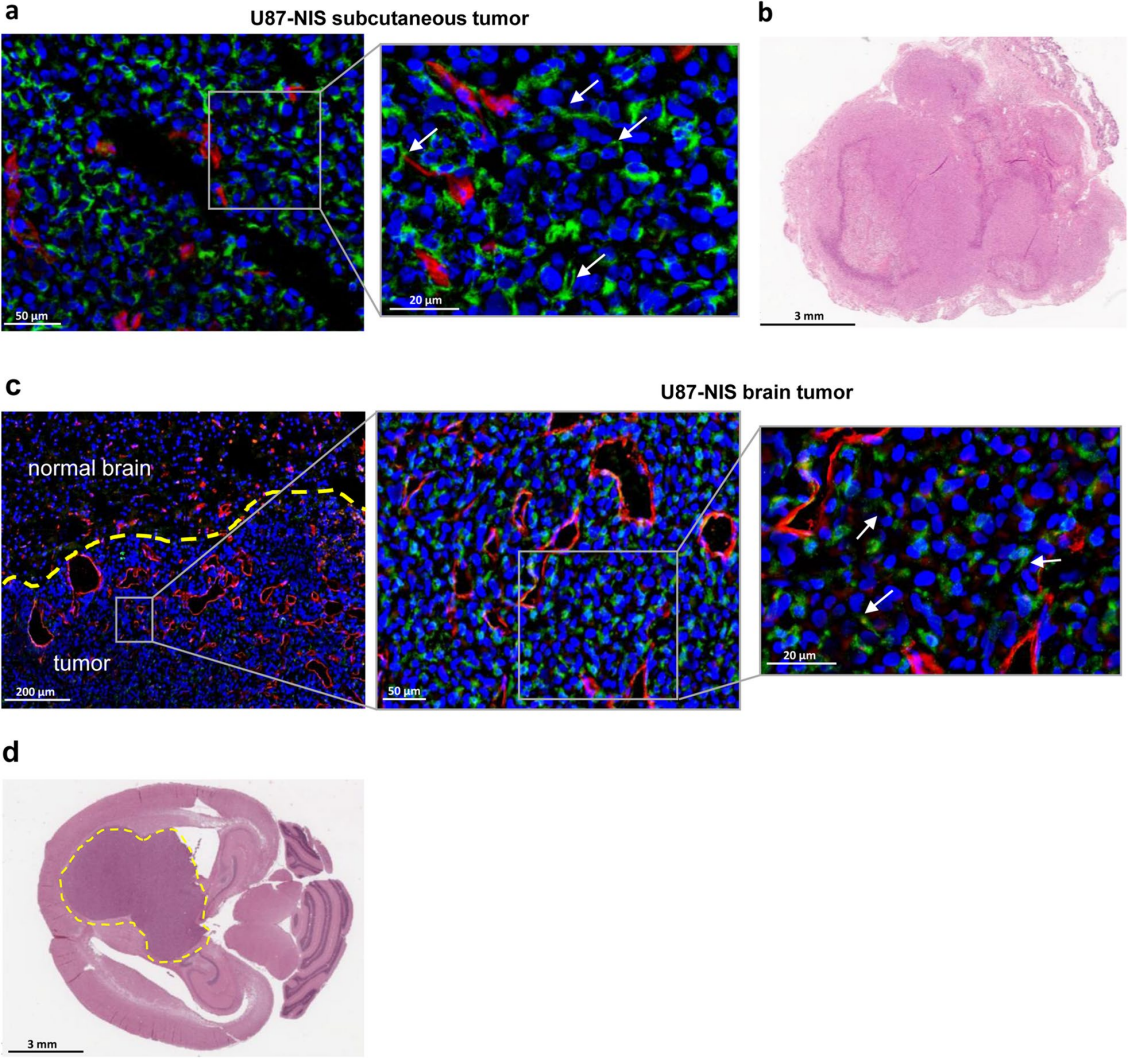
Ex vivo analysis of U87-NIS tumors. **a**, **c** NIS (green) and CD31 (red, labeling tumor vascularization) immunofluorescence staining of cryosections of U87-NIS flank and brain tumors. Nuclei are stained in blue. NIS protein expression is shown at the cellular membrane (white arrows) of the tumor cells.  Increased vascularization of brain tumors is detected as compared to normal brain tissue as well as in contrast to the s.c. model. Section thickness 5 µm (s.c. tumors) and 10 µm (brain section). **b**, **d** H&E of s.c. U87-NIS xenograft tumor and horizontal section of the brain for visualization of the tumor mass. The area of implantation in the right caudate putamen of orthotopic xenografts is shown, the tumor is circled in yellow

To our knowledge no study has reported PET imaging to monitor *NIS* gene expression in brain tumors. The application of the clinically more relevant orthotopic model was then addressed by our group. Nude mice bearing orthotopic U87-NIS brain tumors received [^124^I]NaI or [^18^F]TFB for PET imaging. Both radiopharmaceuticals resulted in NIS-mediated radionuclide accumulation in brain tumors, which was comparable for ^124^I and [^18^F]TFB (Fig. 3a, c). Serial scanning was performed and showed a trend towards an inverse pattern of the accumulated tracers (Fig. 3b, d): Tumoral radioiodide efflux was observed over the scanning time in contrast to the [^18^F]TFB uptake, which showed increasing levels of radionuclide accumulation from the first to the last scan in both settings (s.c. and orthotopic). This increase in [^18^F]TFB uptake over time was also shown in a biodistribution study (dynamic and whole-body static PET/CT scans over 4 h post injection) of healthy participants by evaluation of 15 organs of the human body as well as in the aforementioned hNIS-expressing C6 glioma flank tumor model [46, 47]. Ex vivo analysis of both models, s.c. and orthotopic U87-NIS tumors, showed prominent NIS protein expression localized at the cellular membrane (green, Fig. 4a, c) in dissected tumors, which underlines the NIS-mediated in vivo radionuclide uptake in the tumors with 2D and 3D-imaging devices.

The current data strongly suggest the potential of *NIS* as reporter gene to image brain tumor lesions using PET. Superior imaging by utilization of [^124^I]NaI or [^18^F]TFB as radiotracers allows a detailed/accurate analysis of NIS-mediated radionuclide accumulation in the brain and effectively sets the stage for therapeutic application of [^131^I]NaI. Finally, the image quality of [^18^F]TFB easily produced by most nuclear medicine departments is preferable due to a high signal-to-noise ratio in contrast to lower signal-to-background ratios of [^124^I]NaI-PET. While in these proof-of-principle experimental set-ups ex vivo NIS-transfected glioma cell lines were used, as a next step towards clinical application, we sought to apply and improve upon previously validated methods of systemic non-viral *NIS* gene delivery using [^124^I]NaI or [^18^F]TFB-PET imaging for monitoring of efficacy and tumor selectivity, which will be addressed in the next section.

## Future perspectives: non-viral systemic *NIS* gene delivery to glioblastoma

**Fig. 5:**
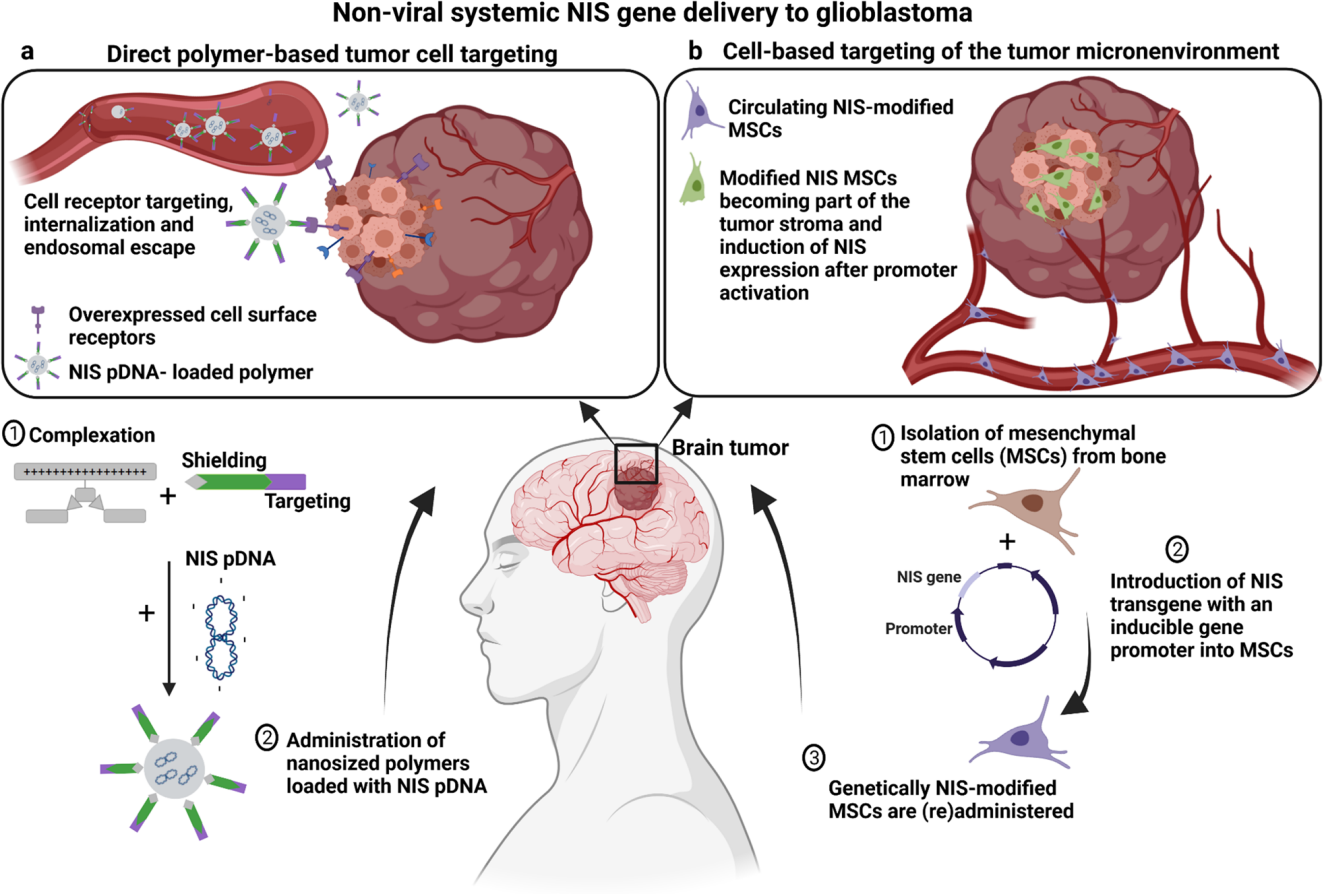
Non-viral systemic *NIS* gene delivery strategies to glioblastoma (GBM). **a** (left panel) Potential approach to use synthetic polymers to deliver the theranostic *NIS* gene directly to GBM cells. (1) The polymer backbone is functionalized with ligands (targeting domain) that have a high affinity to cell surface receptors that are overexpressed in GBM cells. Polymers are loaded with NIS pDNA. (2) Following systemic administration of polymers, the pDNA is released to the GBM cells after binding of the polymer to the cell receptor. **b** (right panel) Mesenchymal stem cell (MSC)-based delivery of *NIS* targeting the tumor microenvironment of GBM. (1) MSCs can be easily isolated from patients from different tissue sources (e.g. bone marrow or adipose tissue) and (2) genetically modified with the *NIS* gene under the control of tumor-stroma specific gene promoters. (3) Engineered MSCs can be amplified in the laboratory and systemically administered back to the patient or over the allogenic barrier. Tumor-secreted factors (e.g. inflammatory cytokines) promote direct migration and extravasation of MSCs to GBM where they become part of the tumor stroma. NIS expression is induced after promoter activation. Following successful *NIS* gene transfer using both delivery platforms, diagnostic and therapeutic application of radioactive NIS substrates can be applied. pDNA; plasmid DNA

The potential of *NIS* as a theranostic gene and the improvement of novel gene delivery systems has expanded the opportunity to use the *NIS* gene therapy concept in extrathyroidal tumors [5, 41]. Based on the gene therapy approaches summarized above, the preclinical development of the *NIS* gene therapy approach will be expanded to other aggressive non-thyroid tumor diseases, such as glioblastoma with the main aim of a clinical phase I/II trial. In ongoing studies we are currently addressing the efficacy of non-viral systemic *NIS* gene delivery systems based on mesenchymal stem cells or synthetic polyplexes to target glioblastoma (Fig. 5) and taking advantage of advanced small animal [^124^I]NaI and [^18^F]TFB-PET imaging [55].

## Conclusion

The application of NIS for radionuclide-based gene therapy of non-thyroidal tumors is a rapidly developing theranostic approach. Non-viral systemic *NIS* transgene transfer systems, such as the use of mesenchymal stem cells or synthetic polymers, have been extensively explored in several preclinical studies, as summarized in this review. These studies have demonstrated the potential of *NIS* as sensitive reporter gene allowing spatial and temporal monitoring of *NIS* transgene expression following therapeutic radionuclide application in non-thyroid cancer settings. The lack of iodide organification in non-thyroid cancer resulting in limited tumoral iodide retention has been raised as an argument against effective *NIS* gene therapy. However, extensive preclinical studies including our own work have convincingly demonstrated that the level of radionuclide accumulation (radioiodide or alternative radionuclides such as ^188^Re and ^211^At) achieved in the tumor, the duration of radionuclide retention, and the distribution of *NIS* transgene expression was sufficient to reach a tumor dose within the range considered sufficient for a therapeutic response in thyroid cancer [56, 57]. More importantly, these levels have been sufficient to elicit a significant therapeutic effect of ^131^I or alternative radionuclides in a variety of tumor models, including clinically highly aggressive tumor models [15–17, 20, 21, 29, 30, 32, 34–37]. For a more detailed discussion of this aspect, we refer to a recently published review paper [5]. The tumor micromilieu might also play a role in regulation of NIS function and/or NIS membrane targeting thereby affecting the efficacy of *NIS* gene therapy approaches, which however has not been explored so far after in vivo* NIS* gene delivery in clinically relevant preclinical tumor models [58]. Based on the extensive data from advanced cancer models including our own data, the *NIS* gene therapy concept should be expandable to disseminated, low volume diseases such as glioblastoma. Low volume disease can be associated with relatively low *NIS* transgene expression. In this instance the high resolution and sensitivity of new imaging modalities should hold much promise for optimizing therapy regimens. Among standard nuclear medicine imaging modalities such as scintigraphy or SPECT, PET offers the highest resolution, sensitivity and allows quantitative measures. The development of [^18^F]TFB as an alternative PET tracer for monitoring NIS biodistribution overcomes many of the issues encountered when using ^124^I. ^18^F-labelled TFB is an excellent iodide analogue with improved imaging quality and availability. In a direct comparison of ^124^I and [^18^F]TFB in a preclinical imaging study—to our knowledge, we were the first to show improved imaging—in an orthotopic hNIS expressing brain tumor model. The results suggest that [^18^F]TFB may serve as promising tracer in the context of NIS-based brain tumor imaging. As a next step, the theranostic features of the *NIS* transgene will be expanded by development of next generation cellular carriers or synthetic polymers to better target the tumor microenvironment of non-NIS-expressing glioblastoma. In this setting, the advantage of [^18^F]TFB for NIS-tracking will facilitate future clinical translation.

## Supplementary Information

**Additional file 1: Fig. S1.** In vitro analysis of U87 cells constitutively expressing the sodium iodide symporter (U87-NIS). **a** Radioiodide uptake was measured in U87-NIS cells and compared to U87 Wt cells at steady-state conditions. U87-NIS cells revealed a 52-fold higher iodide accumulation as compared to U87-NIS cells treated with perchlorate for the blockage of NIS-mediated iodide uptake of the cells. In addition, radioiodide uptake of U87-NIS cells was 102-fold increased in comparison to U87 Wt cells. No iodide uptake above background level was shown in U87 Wt cells. **b** Time course of ^125^I uptake in U87-NIS and U87 Wt cells. Half-maximal levels of perchlorate-sensitive ^125^I accumulation in U87-NIS cells was reached within 5 min and saturation at 45–60 min. **c** NIS-specific immunofluorescence staining of U87-NIS and U87 Wt cells (NIS in red, nuclei in blue). All data are reported as mean ± SEM (**P < 0.01).

## Data Availability

The datasets used and/or analyzed during the current study are available from the corresponding author on reasonable request.

## Abbreviations

ATC: Anaplastic thyroid carcinoma
BBB: Blood brain barrier
CMV: Cytomegalovirus
EBRT: External beam radiation therapy
EGFR: Epidermal growth factor receptor
GBM: Glioblastoma
H&E: Hematoxylin and eosin
HGFR: Hepatocyte growth factor receptor
HIF: Hypoxia inducible factor
HSP: Heat shock protein
ID: Injected dose
i.v.: Intravenous
i.p.: Intraperitoneal
LPEI: Linear polyethylenimine
MSC: Mesenchymal stem cell
NIS: Sodium iodide symporter
pDNA: Plasmid DNA
PEG: Polyethylene glycol
PET: Positron emission tomography
s.c.: Subcutaneous
sg: Salivary glands
SPECT: Single-photon emission tomography
TFB: Tetrafluoroborate
TfR: Transferrin receptor
TGF: Transforming growth factor
TSH: Thyroid stimulating hormone
