# Supplementary figures and images for: The sodium iodide symporter (NIS) as theranostic gene: its emerging role in new imaging modalities and non-viral gene therapy
Source: EJNMMI Res. 2022 May 3;12:25. doi: 10.1186/s13550-022-00888-w (PMC9065223; doi:10.1186/s13550-022-00888-w)

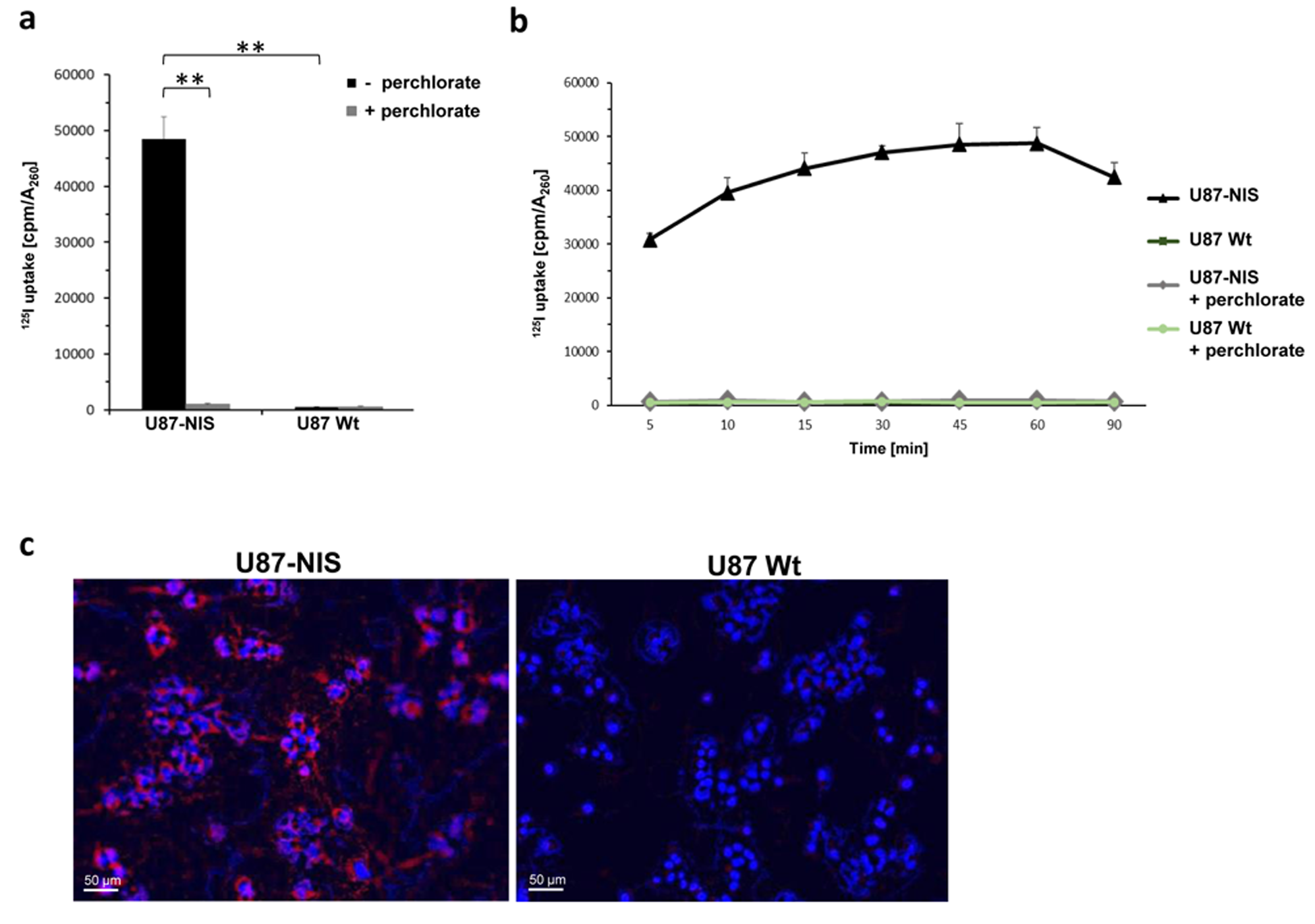

Supplement: Supplementary file 1 — Additional file 1: Fig. S1. In vitro analysis of U87 cells constitutively expressing the sodium iodide symporter (U87-NIS). a Radioiodide uptake was measured in U87-NIS cells and compared to U87 Wt cells at steady-state conditions. U87-NIS cells revealed a 52-fold higher iodide accumulation as compared to U87-NIS cells treated with perchlorate for the blockage of NIS-mediated iodide uptake of the cells. In addition, radioiodide uptake of U87-NIS cells was 102-fold increased in comparison to U87 Wt cells. No iodide uptake above background level was shown in U87 Wt cells. b Time course of 125I uptake in U87-NIS and U87 Wt cells. Half-maximal levels of perchlorate-sensitive 125I accumulation in U87-NIS cells was reached within 5 min and saturation at 45–60 min. c NIS-specific immunofluorescence staining of U87-NIS and U87 Wt cells (NIS in red, nuclei in blue). All data are reported as mean ± SEM (**P < 0.01). [file 13550_2022_888_MOESM1_ESM.tif]
